# Microglia activation mediated by toll-like receptor-4 impairs brain white matter tracts in rats

**DOI:** 10.7555/JBR.32.20170033

**Published:** 2017-12-21

**Authors:** Xinglong Yang, Jingdong Zhang, Lian Duan, Huangui Xiong, Yanping Jiang, Houcheng Liang

**Affiliations:** 1. Department of Neurosurgery, Affiliated Hospital to Academy of Military Medicine Sciences, Beijing 100071, China; 2. Department of Pharmacology & Experimental Neurosciences, University of Nebraska Medical Center, Omaha, NE 68198, USA; 3. Department of Otolaryngology, the 306th PLA Hospital, Beijing 100101, China; 4. Xi'an Bright Eye Hospital, Xi'an, Shaanxi 710000, China.

**Keywords:** lipopolysaccharide, *Rhodobacter sphaeroides*, toll-like receptor 4, microglia activation, white matter tract malfunction

## Abstract

Microglia activation and white matter injury coexist after repeated episodes of mild brain trauma and ischemic stroke. Axon degeneration and demyelination can activate microglia; however, it is unclear whether early microglia activation can impair the function of white matter tracts and lead to injury. Rat corpus callosum (CC) slices were treated with lipopolysaccharide (LPS) or LPS + *Rhodobacter sphaeroides* (*RS*)-LPS that is a toll-like receptor 4 (TLR-4) antagonist. Functional changes reflected by the change of axon compound action potentials (CAPs) and the accumulation of β-amyloid precursor protein (β-APP) in CC nerve fibers. Microglia activation was monitored by ionized calcium binding adaptor-1 immunofluorescent stain, based on well-established morphological criteria and paralleled proportional area measurement. Input-output (I/O) curves of CAPs in response to increased stimuli were significantly downshifted in a dose-dependent manner in LPS (0.2, 0.5 and 1.0 μg/mL)-treated slices, implying that axons neurophysiological function was undermined. LPS caused significant β-APP accumulation in CC tissues, reflecting the deterioration of fast axon transport. LPS-induced I/O curve downshift and β-APP accumulation were significantly reversed by the pre-treatment or co-incubation with *RS*-LPS. *RS*-LPS alone did not change the I/O curve. The degree of malfunction was correlated with microglia activation, as was shown by the measurements of proportional areas. Function of CC nerve fibers was evidently impaired by microglia activation and reversed by a TLP-4 antagonist, suggesting that the TLP-4 pathway lead to microglia activation.

## Introduction

Some professionals, like armed policemen, soldiers, alpinists, boxers, and football players, are prone to mild brain trauma and hypoxia associated with environmental extremes, especially when making an urgent move or intensive training at a high plateaus^[1–5]^. Studies on athletes, patients with ischemic (hypoxic) stroke and animal models show that brain white matter injuries and neuroinflammation, characterized by microglia activation, usually coexist early after trauma and hypoxia exposure^[6–11]^. An electroencephalogram on veterans returning from battlefield showed disrupted connectivity within and between hemispheres, and their neuroimaging found impairment of white matter tracts, such as thalamic radiations and corpus callosum^[12]^. Microglia may be neuroprotective^[13]^; however, more researchers started to study the detrimental effect of activated microglia on white matter function and integrity^[7–9, 11]^. According to some *in vivo* and *in vitro* studies, reagents that inhibit microglia activation can reverse axon injuries induced by mild brain trauma or hypoxia^[14–17]^. This raises a question whether activated microglia *per se* really have an adverse effect on white matter function. To answer this question, we accomplished an experiment about how microglia activation affects white matter tracts, in the absence ofhypoxia or trauma.

Lipopolysaccharide (LPS) is widely used to activate microglia *in vitro* and *in vivo*. Toll-like receptor 4 (TLR-4) is a microglial membrane receptor that mediates its activation^[18–19]^. Periventricular white matter tracts, such as the corpus callosum (CC) and internal capsule, are the brain white matter regions involved in mild brain trauma-related pathophysiological changes. Axon compound action potential (CAP) is widely used to detect the functional impairment of white matter tracts^[20–21]^. In recent years, β-amyloid precursor protein (β-APP) in white matter tracts has been defined as a marker of axon injury as its accumulation reflects the deterioration of fast axonal transport^[22]^. Hence, we used LPS to activate microglia and examined white matter tract injury with the aforementioned approaches.

Morphological criteria for microglial states are clear: ramified cells indicate a resting state; hyper-ramified to amoeboid cells represent a reactive state; non-ramified hypertrophic microglia represent a phagocytic state^[23–25]^. Accordingly, different states of microglia activation display different somata sizes and pseudopodia extension; therefore, measuring proportional areas can evaluate the activation state of microglia/macrophages^[26]^. To monitor and correlate the CC functional impairment with the state of microglia activation, we applied ionized calcium binding adaptor-1 (Iba-1) immunostaining in slices treated equally to electrophysiological recording. Then, we applied a TLR-4 competitive antagonist *Rhodobacter sphaeroides* LPS** (*RS*-LPS**)^[27–28]^, by pre-treatment or co-incubation with CC slices, to explore its effect on CAP input-output responses, β-APP accumulation and microglia activation. We kept a LPS concentration of≤1.0 μg/mL, since LPS doses of>1.0 μg/mL may activate microglia through other pathways^[20]^. With these approaches, we found that the effect of LPS on the function of white matter tract in the CC was correlated with microglia activation via the TLR-4 pathway rather than through some other nonspecific effects of LPS.

## Materials and methods

### Animals

Thirty four adult Sprague-Dawley rats (40–50 days old; 23 males and 11 females) were used in this study, including seventeen for electrophysiological recording, nine for β-APP Western blot analysis, and eight for Iba-1 immunostaining and analysis of proportional areas. Experimental protocols and animal care were observed in accordance with the Guidelines for the Care of Laboratory Animals in Research issued by the Chinese Academy of Military Medical Sciences, which has the equivalent authority to the European Union guideline for Animals Used for Scientific Purpose. The study was approved by Affiliated Hospital of The Academy of Military Medicine Sciences.

### Electrophysiology

Animals were deeply anesthetized with isoflurane and decapitated. Brains were quickly dissected out of the cranial cavity, placed into an ice-cold (4ˆC) oxygenated artificial cerebrospinal fluid (ACSF) and cut into 500 μm slices as previously described^[29]^. The CC slices were treated in ACSF only as a control, and in ACSF containing different doses of LPS (0.1, 0.2, 0.5 and 1.0 μg/mL) for 2.75 to 3.25 hours before recording. When the significant injurious dose of LPS (0.2 to 1.0 μg/mL) was determined, 2.0 μg/mL *RS*-LPS (Invitrogen, Carlsbad, CA, USA) was used to pre-treat CC slices for 1 h. Then, the slices were transferred into LPS (0.5 μg/mL or 0.2 μg/mL) in ACSF for incubation (2.75–3.25 hours before recording). Single use of *RS*-LPS (2.0 μg/mL) was taken as a control. CAP recording was performed as previously described^[29]^. Signals were amplified through an XSP-1 amplifier (Emotiva, Court Franklin, TN, USA) connected to an Axopatch 1D amplifier (Axon Instruments Inc., Union City, CA, USA), digitized at 5kHz with a Digidata 1440A interface (Axon Instruments Inc.) and recorded onto a Lenovo computer through pCLAMP 10.1 software (Axon Instruments Inc.).

### Immunocytochemistry

In the same way, the CC slices were treated for 3 hours for the electrophysiologyical study, but without stimulation and recording. Then, the slices were transferred into 4% paraformaldehyde in 1×phosphate buffered saline (PBS, pH-7.4) and fixed at 4–8ˆC overnight. Then, sections were cryo-protected in sucrose (10%, 20% and 30%). The CC slices were further cut into 14 μm frozen sections and mounted on slides immediately. Sections were routinely blocked and incubated with rabbit anti-Iba-1 (1:300; Wako Chemicals USA Inc., Richmond, VA, USA) overnight. Alexa Fluor conjugated anti-rabbit antibody (1:200, Molecular Probes, CA, USA) was used for immunofluorescent visualization. Control sections were processed in the same way without primary antibody. Slides were sealed with Vectashield with DAPI (Vector Laboratories, Burlingame, CA, USA) and observed under a Nikon-800 microscope. Microimages for proportional areas were acquired through 20× lens and measured by Image J (NIH, Bethesda, MD, USA). Selected area above threshold represented Iba-1-labeled cells. The baseline area was the sum area of 20× images, excluding the area of lateral ventricle.

### Western blotting assays

The prepared brain slices were divided into the ACSF group and ACSF containing LPS 0.2 μg/mL group, both bathed for 3 hours or 6 hours. After an incubation that displayed significant β-APP accumulation, pre-treatment with *RS*-LPS (2.0 μg/mL) followed by incubation with LPS (0.2 μg/mL) or co-incubation with LPS+*RS*-LPS was performed for the same duration. Then, CC tissues were disseced and transferred to Tissue Extraction Reagent 1 (FNN0071, Invitrogen) with 1:1,000 protease inhibitor (P-2714, Sigma-Aldrich, St. Louis, MO, USA), and homogenized. Protein concentration was measured by bicinchoninic acid assay (BCA assay). Routine electrophoresis was carried out using 10% sodium dodecyl sulfate-polyamide gel. Polyclonal rabbit anti-β-APP antibody (1:600; #AB5302, Millipore, CA, USA) was used to detect β-APP. Mouse anti-β-actin (1:10,000, #A2228, Sigma-Aldrich) was applied as a gel loading control. Immunoreactivity bands were detected using enhanced chemiluminescence and developed with autoradiography film. Protein density of β-APP was measured using Image J and normalized to the corresponding β-actin in each sample.

### Statistical analysis

One-way ANOVA analysis of the area under the curve (AUC) was used to examine differences between Input-Output (I/O) curves of CAPs recorded from the control, LPS group and LPS+ *RS*-LPS group. Student's *t*-test was applied to examine differences in β-APP densities between the control and LPS-treated slices. β-APP levels in the control, LPS group and LPS+ *RS*-LPS group were compared with one-way ANOVA. Values of proportional area were processed with one-way ANOVA to compare differences between the control and different doses of LPS, and among control, LPS and *RS*-LPS cases. Statistical analysis was conducted using GraphPad Prism 5 (GraphPad Software Inc. La Jolla, CA, USA).

## Results

### ***RS-LPS*** reversed LPS induced reduction in CAPs

**Fig.1:**
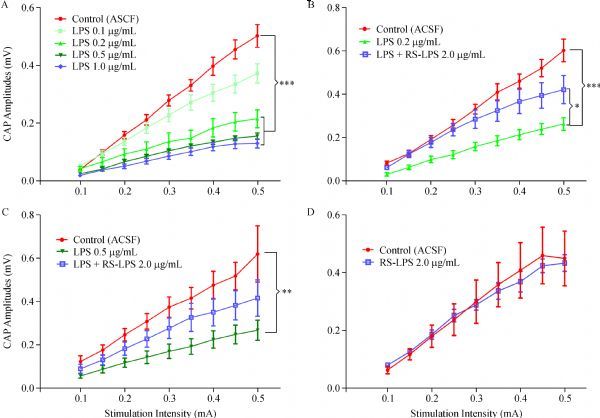
Dose dependent injurious effects of lipopolysaccharide (LPS) treatment on CAPs in corpus callosum (CC) slices and protective effects of ***RS***-LPS pre-incubation. A: Input-Output response curves (I/O) summarized from CC compound action potentials (CAPs) recorded from artificial cerebrospinal fluid (ACSF) (Control), LPS 0.1, 0.2, 0.5 and 1.0 μg/mL treated slices. LPS concentrations from 0.2 to 1.0 μg/mL, but not 0.1 μg/mL, caused significant downshifts of the I/O curve against control, revealed by one-way ANOVA analysis of AUC with Dunnett's posttest to compare all groups to control (*P* < 0.001 in all LPS 0.2, 0.5 and 1.0 μg/mL treated slices, shown as ***). B: Downshift of I/O responsive curve by LPS 0.2 μg/mL incubation was significantly reversed by pre-treatment of CC slices with Rhodobacter sphaeroides LPS(RSLPS) 2.0 μg/mL (one way ANOVA of AUC values with Sidak's posttest: LPS *vs*. LPS + RS-LPS, *P* < 0.05, shown as *; while, the LPS group differed significantly from control (*P* < 0.001, shown as ***). C: downshift of the I/O curve by a higher dose of 0.5 μg/mL LPS was largely reversed by RS-LPS 2.0 μg/mL pre-treatment (one way ANOVA of AUC with Sidak's posttest: LPS vs. LPS + RS-LPS, *P* = 0.092); meanwhile, the LPS group against control was significant different (*P* < 0.01, shown as **). D: Incubation of the CC slices with RS-LPS 2.0 μg/mL alone did not significantly change the area under the I/O curve compared to the control (*P* = 0.962, by two-group *t*-test for area under the curve (AUC)).

The I/O responsive curves of CC fiber CAPs were generated by stimulating fiber bundles (intensity of 0.10–0.50 mA, increment of 0.05 mA) (***Fig. 1***). The amplitude of CAPs was analyzed and measured according to previous methods^[20–21]^. The dose-dependent downshift of I/O curves was recorded and plotted (***Fig. 1A***). One-way ANOVA analysis of AUC revealed that I/O curve downshift by 0.1 μg/mL LPS was not significant, but highly significant by 0.2, 0.5 and 1.0 μg/mL LPS (*P*<0.001, ***Fig. 1A***). However, the reduction of CAPs by LPS was reversed by TLR-4 antagonist *RS*-LPS in a dose-dependent manner. The blocking effect of *RS*-LPS on binding LPS to TLR-4 was evaluated with the ratio of *RS*-LPS to LPS; the vendor's data sheet (www.invivogen.com) stated that a ratio of 100:1 completely blocks TLR-4, and a 10:1 ratio achieves a 90% blocking. Therefore, we chose 2.0 μg/mL *RS*-LPS and 0.2–0.5 μg/mL LPS (*RS*-LPS: 10:1 and 4:1) to explore the effects of *RS*-LPS on LPS-induced I/O curve downshift. One-way ANOVA analysis of AUC values showed that 2.0 μg/mL *RS*-LPS pre-treatment significantly (*P*<0.05) reversed the I/O curve downshift induced by 0.2 μg/mL LPS (***Fig. 1B***); meanwhile, this reversing effect was towards to significant when LPS reached 0.5 μg/mL (***Fig. 1C***, *P* = 0.0915). Finally, compared to the control, AUC values of the slices treated with 2.0 μg/mL RS-LPS alone displayed no significant change (***Fig. 1D***, *P* = 0.962).

### LPS induced microglial activation

**Fig.2:**
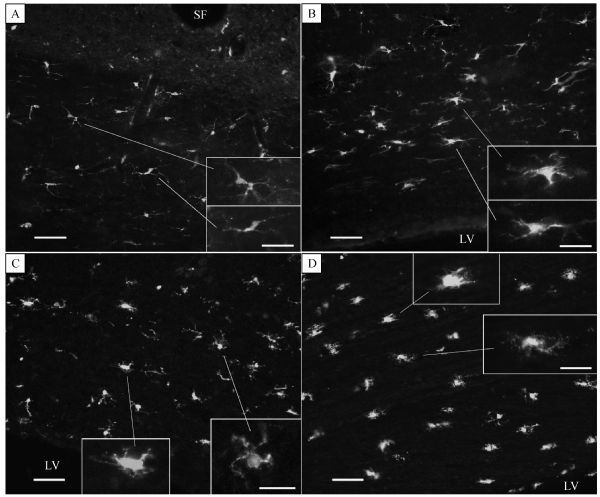
Microglia activation states revealed by immunofluorescent staining of Iba-1 on frozen sections from the CC slices. A: ramified thin-soma microglia in ACSF bathed slices. B: from slices treated with 0.2 μg/mL LPS, in which most of the microglia showed hypertrophic pseudopodia and somata. C: 0.5 μg/mL LPS treatment resulted in hypertrophic somata with shorter bushy pseudopodia and amoeboid like morphology. D: LPS 1.0 μg/mL incubation induced morphological changes similar to those seen in the 0.5 μg/mL LPS treated slices. Scale bars are 100 μm in (A) and (B), 75 μm in (C) and (D), and are 20 μm for all insets. LV: lateral ventricle; SF: sagittal fissure.

**Fig.3:**
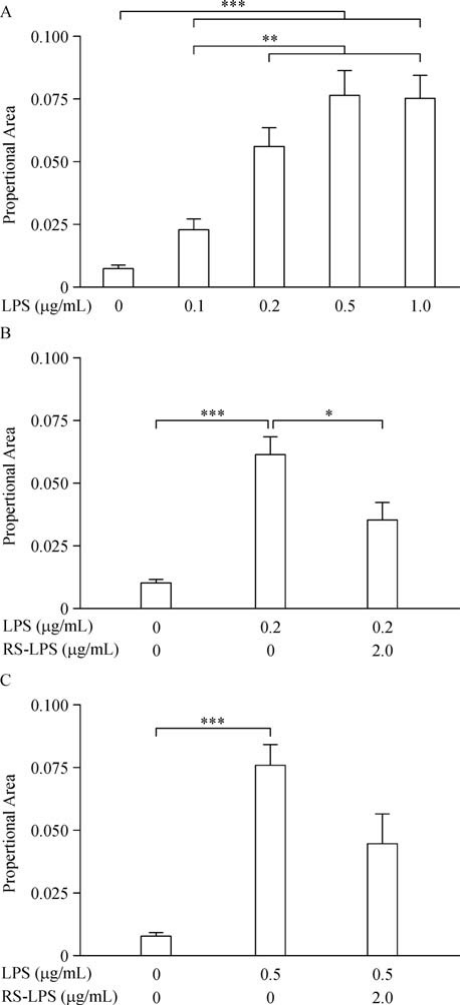
Microglia activation state evaluated by proportional area measurement of positive Iba-1 labeling. A: one-way ANOVA analysis with Dunnett's posttest showed proportional area values measured from LPS treated slices were significantly higher (*P*< 0.001, ***) compared to control; furthermore, that values from slices with LPS of 0.2–1.0 μg/mL treatment were significantly higher (*P*< 0.01,**) *vs*. that of LPS 0.1 μg/mL treated slices. B: when comparing slices incubated in control, 0.2 μg/mL LPS and this LPS plus 2.0 μg/mL RS-LPS pre-treatment, the ANOVA with Sidak's posttest showed that this LPS incubation generated significantly higher proportional area values than the control (*P*<0.001, ***); while RS-LPS pre-treatment significantly reversed LPS-induced increase in proportional area (*P*< 0.05, *) *vs*. this LPS alone treatment. C: in slices incubated in control, 0.5 μg/mL LPS and this LPS plus 2.0 μg/mL RS-LPS pre-treatment, this LPS incubation induced significantly higher proportional area values than the control (*P*< 0.001, ***); however, RS-LPS pre-treatment reversal of the LPS-induced increase in proportional area approached significance (*P* = 0.061).

Resting microglia with thin somata and delicate pseudopodia were observed in the control CC by Iba-1 immunofluorescent staining (***Fig. 2A*** and inset). In the Iba-1 immunostained sections prepared from 0.2 μg/mL-LPS-treated slices, the majority of the Iba-1-positive microglia observed were hyper-ramified with hypertrophic pseudopodia and mild hypertrophic somata (***Fig. 2B*** and inset). Iba-1-positive microglia in the sections from 0.5 and 1.0 μg/mL LPS incubated slices showed hyper-ramified short pseudopodia and overt hypertrophic somata (***Fig. 2C***, ***D*** and insets). Pre-treatment of slices with 2.0 μg/mL *RS-*LPS and LPS incubation resulted in hypertrophic pseudopodia and some mild hypertrophic somata (data not shown). Values of proportional areas^[26]^ were acquired by calculating the selected/whole area ratio of each 20× image. One-way ANOVA analysis indicated a highly significant morphological change evoked by LPS (***Fig. 3A***, *P*<0.001), and a significant increase of proportional area values in LPS 0.2 to 1.0 μg/mL incubated slices versus 0.1 μg/mL bathed ones (***Fig. 3A***, *P*<0.01). Pre-treatment of slices with 2.0 μg/mL *RS*-LPS and 0.2 μg/mL LPS significantly decreased proportional area values compared to that treated with only LPS incubation (***Fig. 3B***, *P*<0.05). The decline of proportional area values by pre-treatment of the same *RS*-LPS concentration followed by 0.5 μg/mL LPS was modest, as expected. The declination was close to the significant level compared to LPS alone treatment (***Fig. 3C***; *P* = 0.061).

### RS-LPS co-incubation attenuated LPS (0.2 μg/mL) induced β-APP accumulation

**Fig.4:**
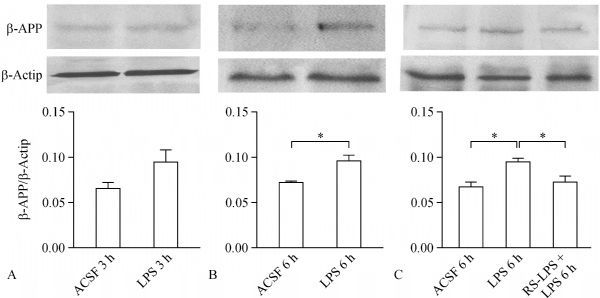
Effects of 0.2 μm LPS with and without ***RS***-LPS 2.0 μm on β-APP axon transport analyzed by Western blotting assays. A: β-APP density was obviously enhanced by 3 hours incubation of LPS, but this did not reach to a significant difference (*P*= 0.089, *t*-test) vs. the control. B: β-APP accumulation was significantly higher in CC tissues vs. the control when duration of LPS treatment was doubled to 6 hours (*P*< 0.05, *,*t*-test). C: treatment of the CC slices with LPS induced significant accumulation of β-APP compared to the control (*P*< 0.05, *, by one-way ANOVAwith Sidak's posttest); meanwhile, co-incubation of LPS and RS-LPS for 6 h significantly reversed β-APP density increment compared to LPS treatment alone (*P*< 0.05, *, with Sidak's posttest).

Since 0.2 μg/mL LPS can induce a significant decrement of CAPs and proportional area values, we selected LPS of this concentration to examine the malfunction of fast axonal transport. After a 3-hour incubation using this LPS, β-APP was evidently accumulated but not to significant level (***Fig. 4A***, *P* = 0.098, Student's *t*-test). We prolonged the incubation duration to 6 hours, and then observed that β-APP density was significantly higher than that in the control (***Fig. 4B***, *P*<0.05). Furthermore, we performed a 6-hour co-incubation using 2.0 μg/mL* RS*-LPS and 0.2 μg/mL LPS. Compared with incubation with single 0.2 μg/mL LPS, *RS*-LPS plus LPS co-incubation significantly reduced the accumulation of β-APP in CC tissues (***Fig. 4C***, *P*<0.05).

## Discussion

The neuroprotection of microglia is clear: as resident innate immune cells in the central nerve system (CNS), microglia protect the brain through fighting against invading pathogens^[30–31]^. A group of authors describe the neuroprotective role of microglia as an ischemic preconditioning^[13]^, since microglia-targeted knockout of receptors in the activation pathway completely blocks this neuroprotection. However, when microglia are repeatedly stimulated, like by repeated episodes of mild brain trauma, they may harm surrounding brain tissues^[8, 32]^. The previous research on microglia activation and white matter damage showed a positive correlation in between^[7–9, 11]^, which can be explained by the fact that degenerating axons or myelin debris recruit macrophages or activate microglia to perform phagocytosis^[33–34]^. However, this study is to address whether microglia activation itself can cause functional impairment of the white matter.

There still lacks evidence that axon malfunction or injury in large white matter tracts is induced by microglia activation through well-established signaling pathways. Microglia activation by LPS (under 1.0 μg/mL) binding through the toll-like receptor 4 (TLR-4) pathway is a well-known model^[18–19]^. In this study, we examined whether microglia activation, in the absence of overt axon damage, hypoxia and myelin debris, could damage the white matter. It is known that functional abnormalities reflect white matter injury^[20–21]^. Thus, we examined CAP changes in CC brain slices treated with LPS of ＜ 1.0 μg/mL, with and without *RS*-LPS (TLR-4 antagonist). Furthermore, β-APP accumulation in axon bundles was examined by β-APP Western blotting assays. Neuronal somata synthesize β-APP and transport it to neuronal terminals through fast axonal transport^[35–36]^. Axon injury dramatically slows down the movement of protein, including β-APP, which finally accumulates in injured white matter tracts^[22, 29, 37]^. In addition, we also monitored microglial morphological changes, quantifying the microglia activation state by measuring the proportional areas^[26]^. Using these methods, this experiment found that *ex vivo* LPS-activated microglia indeed impaired the function of CC nerve fibers, and this injurious effect was correlated with the state of microglial activation.

Compared to the control group, a significant decrement of CAPs, shown by the downshift of I/O curve, was observed in groups treated with LPS of 0.2, 0.5 and 1.0 μg/mL. More importantly, these declines were significantly reversed by pre-treatment with *RS*-LPS (ratio of 4:1 or 10:1 respectively). To evaluate axonal transport, incubation with 0.2 μg/mL LPS for 3 hours did not significantly affect β-APP accumulation. But the 6-hour incubation significantly promoted β-APP accumulation compared to the control group. Furthermore, co-incubation with 2.0 μg/mL *RS*-LPS and 0.2 μg/mL LPS for 6 hours, significantly attenuated β-APP accumulation in the CC tissues compared to tissues treated with single LPS. *RS*-LPS is a polysaccharide analog of LPS, but with one lipid chain less acrylate than LPS^[27–28]^; thus, *RS*-LPS acts as a competitive antagonist blocking the anchoring of LPS onto TLR-4 and preventing the attachment of LPS to this LPS-binding protein. *RS*-LPS is much less toxic than LPS, even when the former's concentration is 10 times of LPS^[27–28]^. That incubation of CC slices with 2.0 μg/mL* RS*-LPS did not induce any functional impairment is consistent with the above reports. This data implied that the CC nerve fiber malfunction is not due to nonspecific interactions between acrylate lipid chains and myelin sheath or axolemma.

The key step of this experiment is to clarify whether these functional changes are correlated with morphological changes in the microglia. So, morphological changes of the microglia in CC slices treated equally to CAPs recording were examined by Iba-1 immunostaining. We found that treating CC slices with 0.2 μg/mL LPS activated the majority of microglia, as indicated by the presence of hyper-ramified pseudopodia and numerous hypertrophic somata. Incubation of slices with 0.5 or 1.0 μg/mL LPS activated all microglia on the slices, as evidenced by the appearance of hypertrophic soma with short bushy pseudopodia and amoeboid morphology. Consistent with these qualitative observations, statistical comparison of proportional area values showed that LPS from 0.2 to 1.0 μg/mL induced significant enhancement of Iba-1 positive proportional area, a measurement that captures the extent of microglial hypertrophic somata and pseudopodia. Compared to LPS, *RS*-LPS pre-treatment also dramatically diminished the proportional area. These findings support the idea that microglia activation through the TLR-4 pathway may lead to the malfunction of CC white matter tract. Previous literature^[7, 32, 38–39]^ suggests that some neurotoxic cytokines from activated microglia might be involved in this malfunction.

This study concludes that: (1) microglia are activated by LPS; (2) activation of microglia by LPS induces CC nerve fiber malfunction in neurophysiology and fast axonal transport; (3) *RS*-LPS attenuates the effects of LPS, indicating that microglial activation through the TLR-4 pathway is responsible for functional changes; (4) the degree of malfunction clearly correlated with the state of microglia activation. However, the mechanism of CC fiber injury is still unknown. Microglia and macrophages cause neuronal pathology primarily through secreting neurotoxic cytokines and/or overactive phagocytosis^[32, 39–40]^. Based on the microglial morphological changes observed in our study, overactive phagocytosis is unlikely to cause functional changes^[23, 32]^. It is highly possible that neurotoxic cytokines lead to axonal injury^[7, 23, 32]^. It also remains to be determined what structures, such as axons, myelin sheath, paranodal or axoglial apparatus, are initially targeted by neurotoxic factors. In cases of mild brain trauma and hypoxia, it is trauma and hypoxia, but not LPS, to provoke the microglia activation.

This study demonstrates that microglia activation *per se* can evoke the detectable malfunction of white matter tracts in the absence of hypoxia or trauma. If microglia are repeatedly irritated till activated by mild head-trauma or hypoxia, white matter injury may ensue. These findings suggest that therapies directed against microglia activation, such as minocycline or ethyl pyruvate or resveratrol serial^[14–17]^, might be a feasible approach to protect at-risk personals from chronic neurological disorders

## References

[1] Bao HX, Chen Z, Lu XL, Effects of rush entry into plateau on recruits' cognitive function[J]. *J Third Mil Med Univ*, 2013, 35: 1498– 1500.

[2] Fu HQ, Yang T, Xiao W, Prolonged neuroinflammation after lipopolysaccharide exposure in aged rats[J]. *PLoS One*, 2014, 9(8): e106331 . 25170959 10.1371/journal.pone.0106331 PMC4149545

[3] Belanger HG, Vanderploeg RD, Sayer N. Screening for remote history of mild traumatic brain injury in VHA: A critical literature review[J]. *J Head Trauma Rehabil*, 2016, 31(3): 204– 214 . 26394295 10.1097/HTR.0000000000000168

[4] Talavage TM, Nauman EA, Breedlove EL, Functionally-detected cognitive impairment in high school football players without clinically-diagnosed concussion[J]. *J Neurotrauma*, 2014, 31(4): 327– 338 . 20883154 10.1089/neu.2010.1512 PMC3922228

[5] Galgano MA, Cantu R, Chin LS. Chronic traumatic encephalopathy: the impact on athletes[J]. *Cureus*, 2016, 8(3): e532 . 27088064 10.7759/cureus.532 PMC4831982

[6] Winston CN, Noël A, Neustadtl A, Dendritic spine loss and chronic white matter inflammation in a mouse model of highly repetitive head trauma[J]. *Am J Pathol*, 2016, 186(3): 552– 567 . 26857506 10.1016/j.ajpath.2015.11.006 PMC4816714

[7] Perez-Polo JR, Rea HC, Johnson KM, A rodent model of mild traumatic brain blast injury[J]. *J Neurosci Res*, 2015, 93(4): 549– 561 . 25410497 10.1002/jnr.23513

[8] Smith C, Gentleman SM, Leclercq PD, The neuroinflammatory response in humans after traumatic brain injury[J]. *Neuropathol Appl Neurobiol*, 2013, 39(6): 654– 666 . 23231074 10.1111/nan.12008 PMC3833642

[9] Suenaga J, Hu X, Pu H, White matter injury and microglia/macrophage polarization are strongly linked with age-related long-term deficits in neurological function after stroke[J]. *Exp Neurol*, 2015, 272: 109– 119 . 25836044 10.1016/j.expneurol.2015.03.021 PMC4591088

[10] Psilodimitrakopoulos S, Petegnief V, de Vera N, Quantitative imaging of microtubule alteration as an early marker of axonal degeneration after ischemia in neurons[J]. *Biophys J*, 2013, 104(5): 968– 975 . 23473479 10.1016/j.bpj.2013.01.020 PMC3870801

[11] Furukawa S, Sameshima H, Yang L, Regional differences of microglial accumulation within 72 hours of hypoxia-ischemia and the effect of acetylcholine receptor agonist on brain damage and microglial activation in newborn rats[J]. *Brain Res*, 2014, 1562: 52– 58 . 24680905 10.1016/j.brainres.2014.03.028

[12] Sponheim SR, McGuire KA, Kang SS, Evidence of disrupted functional connectivity in the brain after combat-related blast injury[J]. *Neuroimage*, 2011, 54(Suppl 1): S21– S29 . 20851190 10.1016/j.neuroimage.2010.09.007

[13] Hamner MA, Ye Z, Lee RV, Ischemic preconditioning in white matter: magnitude and mechanism[J]. *J Neurosci*, 2015, 35(47): 15599– 15611 . 26609155 10.1523/JNEUROSCI.2544-15.2015 PMC4659824

[14] Lorenz P, Roychowdhury S, Engelmann M, Oxyresveratrol and resveratrol are potent antioxidants and free radical scavengers: effect on nitrosative and oxidative stress derived from microglial cells[J]. *Nitric Oxide*, 2003, 9(2): 64– 76 . 14623172 10.1016/j.niox.2003.09.005

[15] Kim HS, Cho IH, Kim JE, Ethyl pyruvate has an anti-inflammatory effect by inhibiting ROS-dependent STAT signaling in activated microglia[J]. *Free Radic Biol Med*, 2008, 45(7): 950– 963 . 18625301 10.1016/j.freeradbiomed.2008.06.009

[16] Zhang F, Liu J, Shi JS. Anti-inflammatory activities of resveratrol in the brain: role of resveratrol in microglial activation[J]. *Eur J Pharmacol*, 2010, 636(1-3): 1– 7 . 20361959 10.1016/j.ejphar.2010.03.043

[17] Haber M, Abdel Baki SG, Grin'kina NM, Minocycline plus N-acetylcysteine synergize to modulate inflammation and prevent cognitive and memory deficits in a rat model of mild traumatic brain injury[J]. *Exp Neurol*, 2013, 249: 169– 177 . 24036416 10.1016/j.expneurol.2013.09.002

[18] Kielian T. Toll-like receptors in central nervous system glial inflammation and homeostasis[J]. *J Neurosci Res*, 2006, 83(5): 711– 730 . 16541438 10.1002/jnr.20767 PMC2440498

[19] Aravalli RN, Peterson PK, Lokensgard JR. Toll-like receptors in defense and damage of the central nervous system[J]. *J Neuroimmune Pharmacol*, 2007, 2(4): 297– 312 . 18040848 10.1007/s11481-007-9071-5

[20] Crawford DK, Mangiardi M, Tiwari-Woodruff SK. Assaying the functional effects of demyelination and remyelination: revisiting field potential recordings[J]. *J Neurosci Methods*, 2009, 182(1): 25– 33 . 19481113 10.1016/j.jneumeth.2009.05.013

[21] Reeves TM, Phillips LL, Povlishock JT. Myelinated and unmyelinated axons of the corpus callosum differ in vulnerability and functional recovery following traumatic brain injury[J]. *Exp Neurol*, 2005, 196(1): 126– 137 . 16109409 10.1016/j.expneurol.2005.07.014

[22] Medana IM, Esiri MM. Axonal damage: a key predictor of outcome in human CNS diseases[J]. *Brain*, 2003, 126(Pt 3): 515– 530 . 12566274 10.1093/brain/awg061

[23] Streit WJ, Walter SA, Pennell NA. Reactive microgliosis[J]. *Prog Neurobiol*, 1999, 57(6): 563– 581 . 10221782 10.1016/s0301-0082(98)00069-0

[24] Kettenmann H. Triggering the brain's pathology sensor[J]. *Nat Neurosci*, 2006, 9(12): 1463– 1464 . 17128280 10.1038/nn1206-1463

[25] Kloss CU, Bohatschek M, Kreutzberg GW, Effect of lipopolysaccharide on the morphology and integrin immunoreactivity of ramified microglia in the mouse brain and in cell culture[J]. *Exp Neurol*, 2001, 168(1): 32– 46 . 11170719 10.1006/exnr.2000.7575

[26] Donnelly DJ, Gensel JC, Ankeny DP, An efficient and reproducible method for quantifying macrophages in different experimental models of central nervous system pathology[J]. *J Neurosci Methods*, 2009, 181(1): 36– 44 . 19393692 10.1016/j.jneumeth.2009.04.010 PMC2737682

[27] Aida Y, Kusumoto K, Nakatomi K, An analogue of lipid A and LPS from Rhodobacter sphaeroides inhibits neutrophil responses to LPS by blocking receptor recognition of LPS and by depleting LPS-binding protein in plasma[J]. *J Leukoc Biol*, 1995, 58(6): 675– 682 . 7499965 10.1002/jlb.58.6.675

[28] Kutuzova GD, Albrecht RM, Erickson CM, Diphosphoryl lipid A from Rhodobacter sphaeroides blocks the binding and internalization of lipopolysaccharide in RAW 264.7 cells[J]. *J Immunol*, 2001, 167(1): 482– 489 . 11418686 10.4049/jimmunol.167.1.482

[29] Zhang J, Liu J, Fox HS, N-methyl-D-aspartate receptor-mediated axonal injury in adult rat corpus callosum[J]. *J Neurosci Res*, 2013, 91(2): 240– 248 . 23161705 10.1002/jnr.23150 PMC3546500

[30] Nimmerjahn A, Kirchhoff F, Helmchen F. Resting microglial cells are highly dynamic surveillants of brain parenchyma in vivo[J]. *Science*, 2005, 308(5726): 1314– 1318 . 15831717 10.1126/science.1110647

[31] Ransohoff RM, Perry VH. Microglial physiology: unique stimuli, specialized responses[J]. *Annu Rev Immunol*, 2009, 27: 119– 145 . 19302036 10.1146/annurev.immunol.021908.132528

[32] Block ML, Zecca L, Hong JS. Microglia-mediated neurotoxicity: uncovering the molecular mechanisms[J]. *Nat Rev Neurosci*, 2007, 8(1): 57– 69 . 17180163 10.1038/nrn2038

[33] Tanaka T, Ueno M, Yamashita T. Engulfment of axon debris by microglia requires p38 MAPK activity[J]. *J Biol Chem*, 2009, 284(32): 21626– 21636 . 19531468 10.1074/jbc.M109.005603 PMC2755886

[34] Smith ME. Phagocytosis of myelin in demyelinative disease: a review[J]. *Neurochem Res*, 1999, 24(2): 261– 268 . 9972873 10.1023/a:1022566121967

[35] Buxbaum JD, Thinakaran G, Koliatsos V, Alzheimer amyloid protein precursor in the rat hippocampus: transport and processing through the perforant path[J]. *J Neurosci*, 1998, 18(23): 9629– 9637 . 9822724 10.1523/JNEUROSCI.18-23-09629.1998 PMC6793291

[36] Kaether C, Skehel P, Dotti CG. Axonal membrane proteins are transported in distinct carriers: a two-color video microscopy study in cultured hippocampal neurons[J]. *Mol Biol Cell*, 2000, 11(4): 1213– 1224 . 10749925 10.1091/mbc.11.4.1213 PMC14842

[37] Li GL, Farooque M, Holtz A, Changes of beta-amyloid precursor protein after compression trauma to the spinal cord: an experimental study in the rat using immunohistochemistry[J]. *J Neurotrauma*, 1995, 12(3): 269– 277 . 7473801 10.1089/neu.1995.12.269

[38] Deng Y, Lu J, Sivakumar V, Amoeboid microglia in the periventricular white matter induce oligodendrocyte damage through expression of proinflammatory cytokines via MAP kinase signaling pathway in hypoxic neonatal rats[J]. *Brain Pathol*, 2008, 18(3): 387– 400 . 18371179 10.1111/j.1750-3639.2008.00138.x PMC8095524

[39] Perry VH, Nicoll JA, Holmes C. Microglia in neurodegenerative disease[J]. *Nat Rev Neurol*, 2010, 6(4): 193– 201 . 20234358 10.1038/nrneurol.2010.17

[40] Neher JJ, Neniskyte U, Brown GC. Primary phagocytosis of neurons by inflamed microglia: potential roles in neurodegeneration[J]. *Front Pharmacol*, 2012, 3: 27 . 22403545 10.3389/fphar.2012.00027 PMC3288722

